# Pesticides and Polychlorinated Biphenyls in Milk and Dairy Products in Croatia: A Health Risk Assessment

**DOI:** 10.3390/foods13081155

**Published:** 2024-04-10

**Authors:** Maja Đokić, Tamara Nekić, Ivana Varenina, Ines Varga, Božica Solomun Kolanović, Marija Sedak, Bruno Čalopek, Darija Vratarić, Nina Bilandžić

**Affiliations:** 1 Laboratory for Residue Control, Department of Veterinary Public Health, Croatian Veterinary Institute, Savska cesta 143, 10000 Zagreb, Croatia; dokic@veinst.hr (M.Đ.); tamara.nekic@gmail.com (T.N.); kurtes@veinst.hr (I.V.); varga@veinst.hr (I.V.); solomun@veinst.hr (B.S.K.); sedak@veinst.hr (M.S.); calopek@veinst.hr (B.Č.); 2 Veterinary and Food Safety Directorate, Ministry of Agriculture of Republic of Croatia, Planinska 2a, 10000 Zagreb, Croatia; darija.vrataric@mps.hr

**Keywords:** milk, dairy products, pesticides, polychlorinated biphenyls, food contaminants, risk assessment, food safety, Croatia

## Abstract

The aim of this study was to evaluate contamination levels and the frequency of detection of organochlorine (OCPs) and organophosphate pesticides (OPPs), pyrethroids, carbamates and polychlorinated biphenyls (seven PCB congeners) in a total of 534 samples of cow’s, sheep’s and goat’s milk and dairy products from Croatia. Concentrations above the limit of quantification (LOQ) were measured for fourteen OCPs, nine OPPs, six pyrethroids, one carbamate and PCBs with a total of 172 results, and no concentrations exceeded the maximal residue levels defined by the European Union. The mean concentrations of pesticides and the sum of seven PCBs were determined in the ranges 0.92–17.4 μg/kg and 1.38–2.74 μg/kg. Pesticides were quantified in 27% of samples, and seven PCBs were quantified in 5.23% of samples. Among the three pesticide groups, the highest numbers of quantified results were found for OCPs (12.1–20.8%). The highest frequencies of detection were found for the sum of 4,4’-dichlorodiphenyltrichloroethane and its isomers (DDTs), hexachlorobenzene and seven PCBs. The sum of seven PCBs was quantified within the range of 3.3–6.67% of samples per milk type and dairy products. Among the OPPs, the highest frequency of detection was found for chlorpyrifos in cow’s milk. Based on the estimated daily intakes, chronic risk characterisation showed no risk for adults or ten-year-old children for the consumption of cow’s milk and dairy products.

## 1. Introduction

Milk is of great importance in human nutrition due to its important essential components. Therefore, ensuring its quality and monitoring its safety is of great importance worldwide. The presence of pesticide residues in food items is considered a major global health issue today. Pesticides most often reach milk and dairy products by feeding cows with contaminated animal feed, grass or corn silage and water, but also as a result of its direct usage in controlling insects and ectoparasites [1,2,3]. Pesticides are significant in resolving problems in agriculture caused by weeds and pests and, thus, ensure productivity and crop protection [4,5]. These chemicals are classified into more than a hundred classes, and the most used today are organochlorines (OCPs), organophosphates (OPPs), carbamates, pyrethroids, neonicophenoxy alkanes and glyphosate-based pesticides [6].

Due to their persistence and bioaccumulation, polychlorinated biphenyls (PCBs) are classified together with OCPs as persistent organic pollutants (POPs) and, since 2001, have been banned worldwide by the Stockholm Convention [7]. Since they are very resistant to degradation in the environment, and given their widespread use in the past, they can still be found in almost all components of the environment and in animals, food and human tissues [8]. Due to their physical and chemical characteristics, PCBs are still used today in numerous industries and are part of numerous general-use items (plastics, rubber products, transformers, heat exchange fluids and others) [9,10]. Therefore, the environment, animals and humans are constantly exposed to these substances [11]. Also, these compounds are characterised by high resistance to and a very slow rate of degradation, causing them to persist in the environment and accumulate in the tissues of living organisms through the mechanisms of the food chain [12,13]. For example, one of the most persistent congeners of PCBs, PCB 153 (2,2′,4,4′,5,5′-hexachlorobiphenyl), has a half-life of 110 years in the environment and 12.4 years in the body [14].

Chronic exposure to pesticides causes negative effects on the endocrine, immune and neurological systems, including kidney and liver problems, respiratory complications and birth defects and is highly susceptible to several human cancers, including head, neck, lung, breast, cervix, prostate, thyroid, brain, colorectal, pancreatic, lung and leukaemia [4,6,15,16,17]. Similar to pesticides, many negative effects on human health have been recorded for PCBs, including reproduction disorders, immune system diseases, neurological disorders and cancers, such as malignant melanoma, non-Hodgkin’s lymphoma and breast cancer [11,14,18]. Considering the established carcinogenic effects, the International Agency for Research on Cancer (IARC) categorises these compounds as carcinogenic to humans (group 1: PCBs and lindane), as probably carcinogenic to humans (group 2A: 4,4’-dichlorodiphenyltrichloroethane (DDT) and dieldrin) and possible human carcinogens (group 2B: chlordane, hexachlorobenzene (HCB), heptachlor and hexachlorocyclohexanes (HCH) [19].

Although OCP and PCB compounds, as already pointed out, have been banned for a number of years, their residues are constantly detected in food, especially food of animal origin, i.e., milk and related products. Numerous studies have shown their residues in milk above the quantification limits of the applied methods and, in some cases, in very high concentrations [2,3,4]. Therefore, the control of pesticides in milk and milk products is important for public health, especially because milk and milk products are consumed to the greatest extent by infants and children and also by the adult population. The acute toxic exposure to pesticides in humans can be well recognised. However, exposure to low doses for a long time by food is not easily observable and is difficult to identify and quantify. Therefore, it is important to carry out the determination of its exposure and assessment of the risk arising from the continuous ingestion of these contaminants in food [4].

The objective of this research was to evaluate the occurrence of pesticides and PCBs in the milk of cows, sheep and goats and dairy products during the period 2015–2022 in Croatia. A second goal was to estimate daily intakes of different age groups of the population and obtain the risk characterisation as to the extent to which consumption contributes to the toxicological reference values.

## 2. Materials and Methods

### 2.1. Sample Collection

A total of 534 samples of milk and dairy products were collected and analysed during the period 2015–2022. Raw milk samples of cows (*n* = 337), goats (*n* = 60) and sheep (*n* = 46) were collected as a part of the National Residue Monitoring Control Plan from dairy farms in the Republic of Croatia. A total of 91 dairy products (cheeses, yoghurts) were collected from retail stores in different Croatian cities. All samples were placed in the freezer at −18 °C until analysis.

### 2.2. Chemicals

Analytical standards of organochlorine (*n* = 23), organophosphorous (*n* = 25), pyrethroids (*n* = 12), carbamates (*n* = 4), pesticides and seven congeners of PCBs (purity 94–99%) were purchased from Dr. Ehrenstorfer LGC Standards (Augsburg, Germany), Toronto Research Chemicals (Toronto, ON, Canada) and Sigma Aldrich (Seelze, Germany). For all pesticides and seven PCBs, the internal standard used was tributyl phosphate, which was provided by Sigma-Aldrich, Seelze, Germany (≥99% purity). Cyfluthrin, cypermethrin, fenvalerate, fluvalinate, tau-, lamda-cyhalothrin, permethrin and resmethrin were reported as the sum of isomers, while deltamethrin was reported as cis-deltamethrin. Acetonitrile (ACN) ULC/MS-CC/SFC purity was obtained from Biosolve Chimie (Dieuze, France), acetone (for HPLC, ≥99.8%) and ethyl acetate (for HPLC, ≥99.7%) were supplied from CHROMASOLV^TM^ (Honeywell Speciality Chemicals Seelze GmbH, Seelze, Germany) and cyclohexane (for HPLC, ≥99.7%) from Sigma-Aldrich Chemie GmbH^®^ (Steinheim, Germany). Dimethylformamide, sodium sulphate (anhydrous) and sodium chloride were obtained from Sigma Aldrich (Bellefonte, PA, USA). Ultrapure water (18.2 MΩ/cm) was obtained from the Milli-Q system (Millipore^®^, Bedford, MA, USA), and nitrogen (N_2_) (5.0 and 5.5 purity was supplied from SOL S.p.A^®^ (Monza, Italy).

### 2.3. Sample Preparation

For the analysis using GC-MS/MS, 50 g of milk or dairy products was measured and placed in a centrifuge bottle. Subsequently, 75 mL of acetone was added and mixed for one minute. An additional 50 mL of the hexane/acetone extraction solvent was then added, and the mixture was blended using a vortex blender for approximately one minute, with the blender blades rinsed with 3 mL of the extraction solvent. The extract was centrifuged at 2500–3500× rpm for 2 to 3 min, and the supernatant was poured through a sodium sulphate column into a tube. Another 50 mL of hexane/acetone extraction solvent was added to the bottom layer, and the process was repeated. The column was rinsed with 20 mL of the hexane/acetone extraction solvent. The eluted fractions were concentrated to 2 mL using a gentle stream of nitrogen at a pressure of 12 ± 2 psi and a temperature of 35 ± 5 °C. The concentrated extract was then transferred to a labelled graduated test tube and adjusted to a volume of 10 mL with a GPC solvent (a mixture of cyclohexane and ethyl acetate in a 1:1 ratio, *v*/*v*).

The cleaning of the test tube was performed using a gel permeation chromatograph (GPC, LC-20 Prominence, Shimadzu, Tokyo, Japan) on the EnviroSep™-ABC preparative (350 × 21 mm) and guard column (60 × 21.2 mm) by Phenomenex (Torrance, CA, USA). The GPC was operated under the following conditions: the mobile phase consisted of cyclohexane and ethyl acetate in a 1:1 ratio (*v*/*v*), with a flow rate of 3 mL/min, detection wavelength set at 254 nm, injection volume of 2 mL, and a collection time ranging from 26 to 47 min. The eluted fractions between 26 and 47 min were collected in a fraction collection tube and concentrated to 1 mL in a concentrator using nitrogen gas at a pressure of 12 ± 2 psi and a temperature of 35 ± 5 °C.

Subsequently, 1 mL of the extract was transferred to a Chem Elut cartridge (Agilent Technologies, Palo Alto, CA, USA) and washed twice with 1 mL of the hexane/acetone solvent, followed by a minimum standing time of 60 min. The Chem Elute cartridge was then positioned above silica and C-18 cartridges, which had been conditioned with 7 mL of hexane-saturated acetonitrile. The sample was eluted from the Chem Elute cartridge using three portions of 6 mL each of hexane-saturated acetonitrile. The eluate was concentrated to 1 mL using nitrogen gas at a pressure of 12 ± 2 psi and a temperature of 35 ± 5 °C. Prior to injection into the GC-MS/MS system, the internal standard was added to every sample and pesticide standard were added to the sample for calibration purposes, employing a matrix-matched calibration approach.

### 2.4. Instrumental Analysis

After sample preparation, the analysis of the pesticide and seven PCBs was performed on an Agilent 7890A gas chromatograph coupled to an Agilent 7000B triple quadrupole mass spectrometer (Agilent Technologies, Palo Alto, CA, USA). An HP-5 ms ultra inert capillary column (30 m × 0.25 mm, 0.25 μm film thickness) containing (5%-phenyl)-methylpolysiloxane (Agilent Technologies, Palo Alto, CA, USA) was used as the chromatographic column.

Chromatographic separation and other operating conditions are presented in a recent study [17]. Briefly, chromatographic conditions were as follows: the injection volume was 2 μL, the inlet temperature was 80 °C until 0.01 min, and then 720 °C min^−1^ to 280 °C; the carrier gas was He at 0.9 mL min^−1^, and the oven temperature program was 70 °C for the first minute, followed by a 25 °C min^−1^ to 150 °C; this then increased from 150 °C to 200 °C for 5 min and finally rose to 280 °C for 13 min. The MS transfer line was at 280 °C, and the ion source was 300 °C. Electron ionisation (EI) at −70 eV was used in MS/MS after 4 min solvent delay. Quadrupole temperatures were 150 °C, collision gas (nitrogen) was 1.5 mL/min and quench (helium) was 2.25 mL/min.

Two or three multiple reaction monitoring (MRM) transitions were selected for each analyte, and the method was divided into 9-time segments with “wide” MS resolutions for all transitions. MassHunter software version number B.07.05.2479 was used for instrument control and data acquisition and processing. The GC-MS/MS optimised parameters for each MRM transition for the 71 compounds are presented in Table 1.

### 2.5. Method Validation

The method’s validation was conducted in compliance with the guidelines outlined in SANTE guidance documents [20,21]. Various analytical parameters were assessed, including linearity, recovery, repeatability, intra-day and inter-day precision, and uncertainty. Linearity was determined by matrix-matched calibration curves in raw milk samples, with eight calibration levels ranging from 0.5 to 200 μg/kg.

Recoveries and repeatability were evaluated at five calibration levels by spiking milk matrix blanks at concentrations of 0.5, 1, 2, 5 and 10 μg/kg. The maximum residue levels (MRL) for milk were set at very low levels, making it important and challenging to achieve a limit of quantification (LOQ) of 0.5 μg/kg. Intra-day precision was assessed by analysing spiked samples over five different days, while inter-day precision was determined by calculating relative standard deviations (RSDs) from the recovery checks of five spiked samples within the same day. Additionally, the uncertainty of the overall procedure was quantified based on the validation data.

### 2.6. Daily Dietary Exposure and Risk Characterisation

The estimation of daily intakes (EDI, μg/kg bw/day) for the pesticide compounds and the sum of seven PCBs in milk and dairy products was conducted using Equation (1):

EDI = C × MS/BW,(1)

where C is the mean concentration of pesticides or PCBs (μg/kg, ww), MS is the meal size (daily consumption of milk or dairy products, gram per day), and BW is the body weight (kg) of adult and child consumers. The EDI was calculated for pesticide and PCB compounds quantified above the LOQ values in more than one sample. The EDI was calculated for cow’s milk as the most commonly consumed type of milk and also for dairy products. The chronic consumption of cow’s milk and dairy products for adult consumers in Croatia was 149.39 g/day, and the average body mass of adult consumers was 76.18 kg [22]. Data on the average consumption of milk in children and adolescents have not yet been published in Croatia. The consumption of milk for ten-year-olds was calculated based on the available literature. Based on the frequency of the consumption of milk and milk products by ten-year-olds in the fourth grade of primary school [23], a daily consumption of 270 g was calculated. The average body weight of a ten-year-old child in Croatia was calculated at 42.28 kg [24].

The chronic risk assessment posed by the pesticides and seven PCBs quantified in milk was assessed by calculating the hazard quotient (HQ) using Equation (2):

HQ = EDI/HBGV,(2)

where HBGV is the health-based guidance value (the toxicological reference values’ acceptable daily intake (ADI), tolerable daily intake (TDI) or reference dose) of the pesticides and seven PCBs used for chronic risk assessment set by the Joint FAO/WHO Meeting on Pesticide Residues [25], the United States Environmental Protection Agency [26] and EFSA [27]. Estimations of HQ < 1 indicate there is no negative effect on consumer health, while HQ > 1 indicates a significant concern for adverse chronic health effects.

### 2.7. Statistical Analysis

Statistical processing of the results was carried out by Statistica 10 (StatSoft^®^ Inc., Tulsa, OK, USA) and Excel (Microsoft Excel, 2010). The concentrations of analysed contaminants in different milk samples and dairy products were calculated as the mean ± standard deviation (SD) and range (minimum and maximum). Statistical processing of the results was performed only for results above the LOQ values. When all results were below the LOQ, <LOQ was indicated.

## 3. Results and Discussion

### 3.1. Method Validation

**Figure 1:**
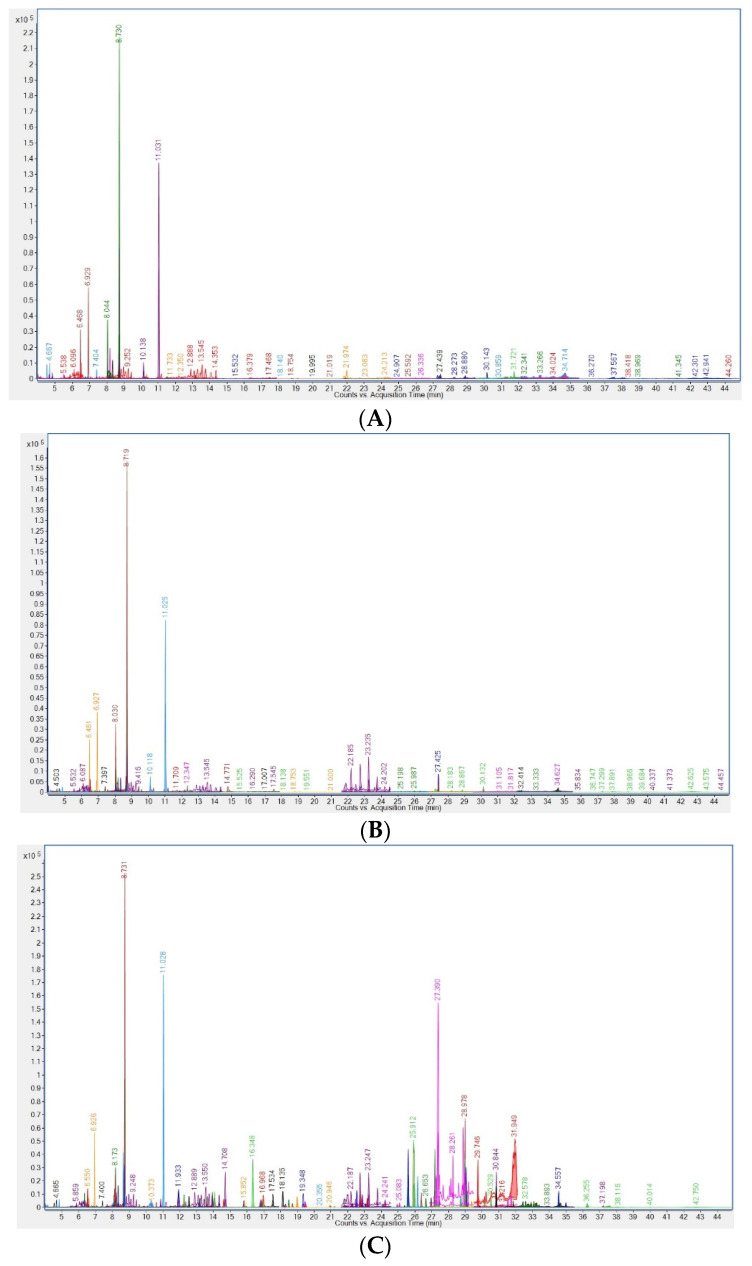
Chromatograms for all optimised analytes in real milk sample (**A**), blank milk sample (**B**) and standard milk sample spiked at LOQ level (**C**).

The parameters of validation for pesticides and seven PCB congeners are shown in Table 2. Chromatograms for all optimised analytes in the real sample, blank milk sample and standard milk sample spiked at the LOQ level are presented in Figure 1.

For the linearity testing of the method, five levels were used. The deviation of the back-calculated concentration from the true concentration was ≤20%. The limits of quantification (LOQ) were determined in accordance with the criteria defined by DG SANTE [20,21], that is, as the lowest spike level at which acceptance criteria were met.

The LOQ values ranged from 0.5 to 10 μg/kg. Recovery and precision at five calibration levels ranged from 82.6 to 118.4%, with an RSD from 7.19 to 19.5%. The obtained results indicated good accuracy and precision.

### 3.2. Occurrence of Pesticide and PCB Residues

Table 3 presents the mean concentrations of OCP and OPP pesticides, pyrethroids and carbamates and seven PCB congeners in the samples of the raw milk of cows, goats, sheep and dairy products collected in Croatia over a period of eight years. The table of results contains only pesticides quantified above the LOQ value. Other pesticides not listed in the table were not quantified (<LOQ). Of the 172 results above the LOQs, none exceeded the maximal residue levels (MRLs) defined by Regulation 396/2005/EC [28]. The mean concentrations of detected pesticides ranged from 0.92 (HCB) to 17.4 μg/kg (carbaryl). Concentrations higher than the LOQ were found out for fourteen OCPs, nine OPPs, six pyrethroids and one carbamate. PCBs are expressed as the sum of seven PCBs, and the sum of the congener numbers are as follows: PCB-28, PCB-52, PCB-101, PCB-118, PCB-138, PCB-153 and PCB-180. Congener PCB-118 was not quantified above the LOQ value in any sample. Pesticides were quantified in 27% of the samples, and seven PCBs were quantified in 5.23% of the samples. The highest frequency of quantified results among the pesticide groups analysed was determined for OCPs, ranging between 12.1% for dairy products and 20.8% for cow’s milk. For HCB and DDTs, the highest detection frequency of 32.4% and 25.5% in the total number of OCP-quantified compounds were found. The mean values for HCB and DDTs were in the ranges of 0.92–1.70 μg/kg and 1.70–3.17 μg/kg.

Concentrations of quantified OCPs in cow’s milk ranged from 1.0 to 3.9 μg/kg with mean values from 0.92 μg/kg (HCB) to 1.78 μg/kg (methoxychlor). Furthermore, the highest percentages of detected OCP results within cow’s milk samples were found for HCB (32.9%), followed by the sum of DDT (DDTs, 30.0%).

Although the usage of OCPs (DDTs, HCB and HCH) has been banned for many years, they are still of great concern because they continue to be present in the environment. DDT can persist in soil for up to three decades [29]. HCB in soil undergoes slow anaerobic and aerobic biodegradation with a half-life of 10.6–22.9 and 2.7–5.7 years [30]. These compounds can accumulate in organisms and be biomagnified through food chains and eventually contaminate food, especially food of animal origin, ultimately reaching the human organism [2,12]. Due to lipolytic properties, OCPs and PCBs accumulate in tissues or food rich in fat [31]. Therefore, food of animal origin and milk and milk products are the main sources of human exposure to these compounds [32].

Numerous studies on DDTs, as the oldest used OCPs, show significant differences in milk concentrations with regard to regions or the level of development of individual countries [2]. Even today, OCPs above the MRL values can be determined in intensive milk production countries and according to the type of agricultural activity. The analysis of milk from dairy farms in Punjab, India, showed three milk samples that exceeded the MRL for DDT [33]. However, mean values for DDT were below 1 μg/kg. In another study conducted on milk produced in five urban areas in different parts of India, the low mean values of HCHs, DDTs, Endosulfan and methoxychlor of 0.09–0.79 μg/kg, 0.24–1.60 μg/kg, 1.21–1.24 μg/kg and 0.24 μg/kg, respectively, were determined, with concentrations above the MRL in the ranges of 18–85 μg/kg, 13.4–170 μg/kg, 86–130 μg/kg and 40 μg/kg, respectively [4]. However, in Ethiopian cow’s milk, DDT concentrations up to 10 times higher than the prescribed MRL values were measured (269–477 μg/kg, [34]; 243–420.80 μg/kg, [35]). Also, significantly higher concentrations of DDTs (113.40–218 μg/kg) compared to the MRL were determined in cow’s milk from urban areas in Romania with varying intensity of agriculture activities [36].

Research in Poland showed concentrations of DDTs, HCHs and heptachlor in the ranges 1.72–3.75 μg/kg, 0.72–4.031 μg/kg and 0.086–2.854 μg/kg, respectively [37]. Within the Total Diet Study (TDS) conducted in Hong Kong, low concentrations of DDTs and HCB (0.7 and 0.0 ug/kg) were measured in dairy products [38]. Also, very low concentrations of HCB (0.015 μg/kg) were determined in Catalonia, Spain [39]. DDTs and HCH concentrations found in milk from Nanjing, China, were below the maximum residue limits (MRLs) with mean levels of 2.25 μg/kg and 0.6 μg/kg, respectively [12]. A recent study from northern China showed low levels of HCHs (0.07 μg/kg) and DDTs (0.1 μg/kg) in cow’s milk [40]. However, analysis conducted at the national level in China and based on published studies showed mean values of 6.1 μg/kg and 5.3 μg/kg for DDTs and HCHs in dairy products [41].

In 97.4% of milk samples from the Greek market, at least one DDT isomer was detected, while DDE was the most frequently detected in 94.9% of samples, though all concentrations were below 1 μg/kg [42]. Studies from Pakistan showed high values exceeding the MRLs for α-endosulphan and β-endosulphan (112.69 and 107.16 ug/kg; [43]), and the sum of endosulfan was 118.9 ug/kg in cow’s milk [44]. In contrast, concentrations of total endosulfan in a recent study in China were below the measurable limits [40].

In the present study, sheep’s and goat’s milk had the highest frequency of detected compounds above the LOQ value (41.3 and 36.7%), and OCPs were detected in 20 and 19.7% of the total sheep’s and goat’s milk samples. The mean values of OCPs in sheep’s and goat’s milk ranged from 1.03 to 2.30 μg/kg. Few studies are available with the results of pesticide determination in these two types of milk. Unlike this study, in the milk of sheep and goats from Greece, no OCP or OPP residues were measured [45]. Also, analyses of OCPs (DDT, heptachlor, endosulfan, etc.) in organic and conventional goat’s milk from Indonesia [46] and goat’s milk from Nigeria [47] showed concentrations below the limits of detection. However, a study from Turkey showed high levels of OCPs in sheep’s milk with concentrations of 122.98 μg/L for beta-HCH, 4.49 μg/L for HCB, 16.34 μg/L for 4,4′-DDT and 26.09 μg/L for methoxychlor [48].

Today, insecticides make up about 20% of the world’s pesticide market, while OPPs are the most widely used insecticides [49]. Although they are neurotoxic like OCPs, they are considered a more favourable alternative because their levels of bioaccumulation in organisms are much lower compared to OCPs. They are characterised by a wide spectrum of action against pests due to their rapid action, i.e., immediate neurotoxicity against a large number of target organisms [50]. Another advantage of their use in agriculture is their cost efficiency [51]. Commonly used OPP insecticides in agriculture are diazinon, chlorpyrifos, chlorpyrifos-methyl, dichlorvos, fenithrotion, parathion, methyl-parathion, malathion, azinphos-methyl, phosmet and tetrachlorvinphos [50].

In this study, the frequent quantification of OPPs in the total number of quantified results in the raw milk of cows, goats, sheep and dairy products ranged between 3.3 and 10%. The mean values of OPP compounds ranged from 1.25 μg/kg for bromophos-ethyl determined in goat’s milk to 3.65 μg/kg of diazinon in sheep’s milk. The highest percentage of detected results of an individual compound was found for chlorpyrifos (47.4% among OPPs) in cow’s milk.

A study from Brazil showed chlorpyrifos detected in the range of 0.06–5.85 μg/L in cow’s milk [52]. Concentrations above the MRL, the maximal level of 45.4 μg/kg and mean of 2.58 μg/kg for chlorpyrifos, were determined in the milk from dairy farms in Punjab, India [33]. On the other hand, ethion levels of 0.92 μg/kg were measured, and the mean values of chlorpyrifos and ethion of 0.76–1.71 μg/kg and 0.68–1.46 μg/kg were obtained in a study of milk from an area adjacent to five cities in India [4]. Within the TDS on pesticide residues in France, primiphos-methyl was measured at maximal levels of 30 and 10 μg/kg in dairy-based yoghurt and ultra-fresh dairy-based desserts [53]. High concentrations of chlorpyrifos were determined in goat’s (0.76 mg/L) and sheep’s milk (0.69 mg/L) from Pakistan [54]. In a recent study from China, no OPPs were quantified in cow’s or sheep’s milk [55].

Pyrethroids are widely applied today and may be found in many commercial insecticide formulations used for insect control on food crops (maize, rice, sunflower, sweet corn and others) to prevent insect infestation in empty grain containers or on cereals before storage in breeding tanks, but also during livestock breeding for insect control on farms [56,57].

In the present study, pyrethroids and carbamates were measured in a range of 1.2 (bifenthrin in cow’s milk) to 27.9 μg/kg (carbaryl in sheep’s milk) but were not detected in dairy products. A recent study in Serbia showed that only 11.4% of analysed milk samples had pesticide residues above the LOQ, and among them, bifenthrin was measured at concentrations of 23 and 19 μg/kg [5]. A study from Brazil showed the presence of cypermethrin, deltamethrin and bifenthrin in the range of 0.02 to 1.61 μg/L [52]. A second study in Brazil also found low mean pyrethroid concentrations (0.02–0.08 μg/L) [56]. A study of dietary pyrethroid exposures in adults from North Carolina found bifenthrin (7.33 μg/kg), esfenvalerate (16.7 μg/kg) and *cis*-permethin (0.33 μg/kg) in yoghurt [57]. Permethrin and cypermethrin were measured in milk from areas near five cities in India, with a mean range of 0.39–1.74 μg/kg and 0.30–28.17 μg/kg, respectively, and maximum levels (exceeding the MRL) of 340 μg/kg and 1840 μg/kg, respectively [4].

Regarding pyrethroids and carbamates in sheep’s and goat’s milk, the highest mean value of 17.4 μg/kg was determined for carbaryl in sheep’s milk. A study on goat’s and sheep’s milk from Pakistan showed maximal concentrations of bifenthin at 0.71 and 0.72 mg/L and deltamethrin at 0.71 and 0.80 mg/L, respectively [54].

Although most studies have shown exposure to PCBs through fish consumption, dairy products also contribute to exposure to PCBs because they are more often used in food [10,58]. Studies have shown that almost 99% of PCBs are introduced to lactating cows through feed, with negligible contamination by air and water. According to the efficiency of absorption of PCBs after the intake of these contaminants, the highest value of PCBs that transfer from the gastrointestinal tract to milk occurs after 2–3 days [8].

In the present study, a frequency detection of seven PCBs from 3.3% (dairy products) to 5.63% (cow’s milk) was determined. Seven PCBs were calculated in the range from 1 to 25.6 μg/kg (highest in cow’s milk). Mean concentrations in milk samples of cows, sheep, goats and dairy products ranged from 1.38 to 2.85 μg/kg (highest in dairy products). The mean concentration for the sum of seven PCBs of 2.74 μg/kg was largely contributed to by the maximum determined value of 25.6 μg/kg.

According to the database for PCB levels in milk from different European countries, a mean PCB value of 10.7 μg/kg was calculated [32,59]. The evaluation of data on concentrations of PCBs sampled in the period 1995–2010 from 24 European Union countries, including Norway and Iceland, showed a trend of decreasing levels of PCBs in the food group “raw milk and milk products” [60]. In recent studies from Italy, the results of a reduction in PCB levels in milk over the observed years were found [61,62]. Since the values for PCBs in this research are expressed based on fresh weight and not on a lipid basis, a comparison was made with studies on PCB concentrations expressed in the same way. It should be emphasised that a lack of determination of the percentage of fat in the samples is a limitation of this research, especially because milk and milk products are eminently fatty matrices, and a good part of the compounds analysed are of a lipophilic nature.

PCBs determined in cow’s milk in France within the Total Dietary Study (TDS) showed levels below 1 μg/kg (44 ng/kg) [58]. In cow’s milk from California, congeners PCB-101, PCB-118 and PCB-138 were detected at low concentrations, below 0.03 μg/L [63]. The concentration of PCBs below 1 μg/kg was measured in milk from northern China [40] but also within the national analysis of data for PCBs available at the national level for China [41]. The content of seven PCB congeners in milk collected from three different areas of Slovakia was found to be less than the LOQ values [64]. However, significantly higher PCB concentrations (9.48 μg/kg) were determined in a recent study from southern Italy [65]. In cow’s milk from Bangladesh, the mean values of PCB congeners were measured in the range from 0.52 to 3.35 μg/kg [10].

In conclusion, according to the results determined in this paper, a similar trend between Croatia and other EU countries can be observed [32,37,39,58,59]. This is to be expected, considering that the production and use of the vast majority of OCPs are banned in Europe. Also, the use of these contaminants is significantly reduced throughout the world, but in some countries, such as India, the use of significant amounts of DDTs is permitted for the control of malaria [4,42]. The world map of pesticide use for significant agricultural problems in weed and pest control shows great differences [6]. Environmental burdens of pesticides vary, and thus, their residues in different food items, such as milk and dairy products, also vary in different regions of the world, as shown through the comparison of this study’s results with results from other countries.

### 3.3. Dietary Exposure and Risk Assessment

Table 4 presents the dietary exposure and potential chronic health risk to pesticides and seven PCBs associated with the consumption of milk and dairy products for adults and children (ten-year-olds) in Croatia. Risk assessments were carried out for cow’s milk and dairy products, mostly consumed in Croatia, while goat’s and sheep’s milk are not often consumed. This was confirmed by the fact that Croatia produced 405,525 t of cow’s milk in 2022, and only 2231 t of sheep’s and 3556 t of goat’s milk [66].

Daily intakes (EDIs) of OCPs calculated for the consumption of cow’s milk for adults varied in the range of 1.80–3.49 ng/kg bw/day, while for children, this was 5.88–11.4 ng/kg bw/day. For dairy products, the highest EDI values were calculated for DDTs, 6.21 ng/kg bw/day for adults and 20.3 ng/kg bw/day for children. For OPPs, pyrethroids and carbamates, EDI values in milk and dairy products were determined in the ranges of 2.55–4.45 ng/kg bw/day for adults and 8.31–14.5 ng/kg bw/day for children. Similar EDI values for cow’s milk and dairy products were determined for PCBs for both adults (5.37 and 5.59 ng/kg bw/day) and children (17.5 and 18.2 ng/kg bw/day, respectively).

Higher dietary exposure to DDTs, endosulfan HCB and HCH via milk consumption compared to the results in this study was found in studies from Hong Kong and China [12,38]. Significantly higher values of dietary exposure by milk consumption for DDTs and HCH were measured for adults (29.13 and 12.62 ng/kg bw/day) and children (69.52 and 30.45 ng/kg bw/day) in Nanjing, China [12]. High EDI values of 23.8 and 39.9 ng/kg bw/day for DDTs and 8.5 and 16.6 ng/kg bw/day for endosulfan were found for the average population of Hong Kong [38].

A study from Turkey showed lower EDI values for DDTs for age groups of adults and children (2.59 and 6.75 ng/kg bw/day), the sum of endosulfan (1.86 and 4.83 ng/kg bw/day) and metocychlor (0.43 and 1.13 ng/kg bw/day), but higher values for HCHs (4.78 and 12.44 ng/kg bw/day) [67] than those determined in this study. A similar EDI for HCB was found (2.03 ng/day) through the consumption of milk determined in this study in Catalonia, Spain [39].

The determined values of EDIs for seven PCBs in this study are in line with the mean exposure to the sum of PCBs (six NDL-PCB indicators) found in EU countries between 4.3 and 25.7 ng/kg bw/day [60]. Very low EDIs were determined for PCBs within the TDS conducted in France, with 0.046 ng/kg bw/day for adults and 0.225 ng/kg bw/day for children [58]. The study also showed that milk and dairy products contribute only 5.9 and 5.8% to the total exposure of children to PCBs compared to other types of food (for example, meat contributes 7.9% and fish contributes 47.9%). The comparison of PCB exposure by different food types with previous research in France from 2007 shows that the contribution of meat has decreased while the dairy product contribution has remained stable [58]. Estimated dietary exposure to PCBs in Austria was 3.37 ng/kg bw/day for children and 2.64–3.19 ng/kg bw/day for adults. Within that study, milk and dairy products were the food groups contributing most (50–55%) to the total dietary intake of PCBs in adults and children [68]. Lower EDI values for PCBs in adults and children (1.14 and 2.97 ng/kg bw/day) were determined in milk from Turkey [67]. In the scope of the TDS in Australia, EDI values for adults and children in the age group between 2 and 17 years were calculated within the ranges of 0.015–2.3 ng/kg bw/day and 0.0032–3.9 ng/kg bw/day [69].

**Table 4:** The estimation of daily intake and risk characterisation of pesticides and seven PCBs in cow’s milk and dairy products.

Active Substances	HBGV (ng/kg bw/day)	Cow’s Milk				Dairy Products			
		EDI (ng/kg bw/day)		HQ		EDI (ng/kg bw/day)		HQ	
		Adults	Children ^f^	Adults	Children ^f^	Adults	Children ^f^	Adults	Children ^f^
OCPs									
Chlorobenzilate	20,000 ^a^	2.55	8.31	1.27 × 10^−4^	4.15 × 10^−4^				
∑DDT	10,000 ^b^	3.33	10.9	3.33 × 10^−4^	1.09 × 10^−3^	6.21	20.3	6.21 × 10^−4^	2.02 × 10^−3^
∑endosulfan	6000 ^a^	3.30	10.7	5.49 × 10^−4^	1.79 × 10^−3^	3.82	12.5	6.37 × 10^−4^	2.08 × 10^−3^
∑HCH	2000 ^d^	2.04	6.65	1.02 × 10^−3^	3.32 × 10^−3^				
Heptachlor	100 ^b^					2.16	7.03	0.022	0.070
Hexachlorobenzene	170 ^c^	1.80	5.88	0.011	0.035	2.51	8.18	0.015	0.048
Methoxychlor	5000 ^e^	3.49	11.4	6.98 × 10^−4^	2.27 × 10^−3^				
OPPs									
Bromophos-ethyl	3000 ^b^	2.65	8.63	8.82 × 10^−4^	2.87 × 10^−3^	4.45	14.5	1.48 × 10^−3^	4.84 × 10^−3^
Chlorfenvinphos	500 ^b^	2.55	8.31	5.10 × 10^−3^	0.017				
Chlorpyrifos	1000 ^a^	2.92	9.52	2.92 × 10^−3^	9.52 × 10^−3^				
Ethion	2000 ^b^	4.32	14.1	2.16 × 10^−3^	7.03 × 10^−3^				
Pirimiphos-methyl	4000 ^a^	2.74	8.95	6.86 × 10^−4^	2.23 × 10^−3^				
P&C									
Bifenthrin	15,000 ^a^	2.74	8.95	6.86 × 10^−4^	2.23 × 10^−3^				
PCBs									
∑PCB	20 ^d^	5.37	17.5	0.27	0.88	5.59	18.2	0.28	0.91

HBGV—Health-based guidance values. EDI—Estimated daily intake (ng/kg bw/day). HQ—Hazard quotient. ^a^ Acceptable daily intake, ADI [27]. ^b^ Acceptable daily intake, ADI and provisional tolerable daily intake, PTDI [25]. ^c^ Tolerable daily intake, TDI [30]. ^d^ Reference dose [26]. ^e^ Tolerable daily intake, TDI [70]. ^f^ Ten-year-old children.

For chronic risk assessments in this study, HQs were determined for adults and children by the ratio of EDI values to the corresponding health-based guidance values (HBGV) defined by safety agencies and European or international bodies (Table 4). For all analysed compounds, an HQ < 1, which is lower than the HGBV, was determined. Furthermore, for all OCPs, OPPs and bifenthrin, the calculated values were significantly lower than 0.02. In the case of the sum of seven PCBs, similar HQs were calculated for both cow’s milk and dairy products for adults (0.27 and 0.28) and children (0.88 and 0.91, respectively). Based on this risk evaluation, possible negative and adverse effects on the health of adults and children regarding the use of cow’s milk and dairy products in the diet can be excluded.

Examples of different HQ values and, accordingly, different concerns for the chronic toxic effects of certain pesticides were determined in a study in Turkey [67], where HQs < 1 for adults and children were found for DDTs, endosulfan and methoxychlor, but HQ > 1 was found for heptachlor and PCBs. Extremely high HQs of 16 and 41.46 were found for HCHs.

A limiting factor of this study is the disadvantage of comprehensive data from the literature on the dietary intakes of milk and dairy products by the Croatian population for infants (up to 12 months old), toddlers (13–36 months old), children (37 months–9 years old) and adolescents (up to 10 years old). An additional limitation is the unavailability of data concerning the distribution of body weight in those age groups. Therefore, this study could not evaluate the exposure that the most sensitive population groups (infants and children up to 10 years old) have to these compounds who consume the largest amounts of milk and could be identified as having an increased risk of exposure with potentially long-term toxic effects.

## 4. Conclusions

In this study, residues of OCPs, OPPs, pyrethroids, carbamates and PCBs were determined in the milk of cows, sheep, goats and dairy products over a period of eight years. Pesticides were quantified in 27% and seven PCBs in 5.23% of samples. The quantification of 71 compounds in the milk of cows, sheep and goats and dairy products revealed levels above the LOQ values for fourteen OCPs, nine OPPs, six pyrethroids, one carbamate and the sum of seven PCBs, and no concentrations exceeded the MRL values defined by the EU legislation. The highest frequency of determination was found for OCP compounds. Among the total quantified compounds of OCPs, the highest frequencies of detected results were found for HCB and DDTs. Among OPPs, the highest percentage of detected results was found for chlorpyrifos in cow’s milk.

Based on the quantified concentrations of pesticides and seven PCBs in cow’s milk and dairy products, the estimated daily intakes of these compounds were calculated for ten-year-old children and adults in the Croatian population. Considering that the contaminant levels in samples are lower than the EU limits and risk characterisation shows results below HBGV, it can be concluded that dietary exposure to these compounds may not pose a risk to consumers’ health.

## Figures and Tables

**Table 1:** GC-MS/MS optimised parameters.

Compound	RT (min)	Ion Precursor; Ion Product 1 (*m*/*z*)	CE * (eV)	Ion Precursor; Ion Product 2 (*m*/*z*)	CE * (eV)	Ion Precursor; Ion Product 3 (*m*/*z*)	CE * (eV)
Organochlorine pesticides (OCPs)							
Aldrin	23.22	262.8; 193.1	40	262.8; 191.1	40		
Chlordane, cis-	26.68	374.7; 266.0	27	374.7; 302.9	10		
Chlordane, trans-	26.09	374.8; 266.0	30	374.8; 268	22		
Chlorobenzilate	12.20	139.1; 111.0	16	139.1; 75.1	22		
p,p′-DDD	28.95	234.8; 165.1	27	234.8; 199.1	20		
p,p′-DDE	27.51	246.0; 175.2	40	246; 176.1	10		
o,p′-DDT	29.06	234.9; 165.0	27	234.9; 199.1	17		
p,p′-DDT	30.13	234.9; 165.1	25	234.9; 199	20		
Dieldrin	27.55	262.7; 192.9	37	262.7; 191	35		
alpha-endosulfan	26.58	240.9; 206.1	15	240.9; 171	30		
beta-endosulfan	28.66	241.0; 206.0	15	238.8; 204	15	195; 159	5
endosulfansulfate	30.03	271.9; 236.9	10	271.9; 116.9	40		
Endrin	28.33	262.7; 193.1	35	262.7; 191.1	35		
alpha-HCH	15.78	218.9; 183.0	7	218.9; 181	7		
beta-HCH	17.12	218.9; 183.0	6	218.9; 180.8	6		
gamma-HCH/Lindan	17.46	218.9; 183.0	5	218.9; 181.0	5		
Heptachlor	21.35	271.6; 237.0	15	273.7; 239	15		
Heptachlorepoxid, egzo-	25.10	352.8; 263.0	20	352.8; 281.9	20		
Heptachlorepoxid, endo-	25.29	252.8; 183.1	40	252.8; 181.2	40		
Hexachlorobenzene	16.17	283.9; 213.9	32	283.9; 248.9	32		
Methoxychlor	14.07	227.0; 141.1	10	227.0; 169.1	12		
Pentachloroaniline	19.94	264.7; 194.0	28	264.7; 203	28		
Quintozene	17.72	236.9; 118.9	30	236.9; 142.7	30		
Organophosphorus pesticides (OPPs)							
Azinphos-ethyl	33.85	160.0; 132.0	0	132.; 104.0	4	159.9; 104.9	12
Bromophos-ethyl	26.26	358.8; 303.0	15	358.8; 330.9	5		
Carbophenothion	29.78	156.9; 74.9	40	156.9; 121.1	25		
Chlorfenvinphos	25.54	266.9; 159.0	15	266.9; 81	30		
Chlorpyrifos	23.68	314.0; 258.1	7	314; 286.1	5		
Chlorpyrifos-methyl	21.00	285.7; 93.0	20	287.7; 93.0	20		
Diazinon	18.43	199.0; 92.9	18	199; 135,1	10		
Dichlorvos	7.37	109.0; 78.7	5	184.9; 93	10		
Ethion	29.12	230.8; 128.9	25	230.8, 175	10		
Fenchlorphos	21.83	284.7; 269.8	15	284.7; 93	30		
Fenithrotion	22.57	276.8; 260.0	5	277; 125.1	20	277; 109.1	20
Fenthion	19.30	278.0; 109.1	20	278.0; 124.9	20		
Malaoxon	17.23	127.0; 98.8	20	127.0; 82.9	20		
Malathion	23.17	173.0; 98.8	15	173.0; 117.2	10	158.0; 125.0	8
Methidathion	26.18	145.0; 85.0	5	145; 58.1	15	302; 85	16
Mevinphos	9.80	127.0; 109.1	11	192; 127	11		
Paraoxon-metyl	14.69	230.0; 200.1	5	230.0; 136.1	5		
Parathion-ethyl	23.70	291.0; 109.0	10	291.0; 81.0	25		
Parathion-methyl	21.00	262.8; 109.0	9	263; 246	2	262.8; 79.1	30
Pirimiphos-methyl	18.45	290.0; 124.9	25	290.0; 151.0	20		
Profenofos	27.39	208.0; 63.1	44	338.9; 269.0	12		
Propetamphos	17.77	138.2; 109.9	5	138.2; 64.2	15		
Pyrazophos	33.70	220.9; 193.1	10	220.9; 149.1	10		
Tetrachlorvinphos	26.59	330.8; 109.0	20	330.8; 79.0	27		
Triazophos	29.54	161.0; 134.0	5	161.0; 106.0	10	257.0; 162.0	5
Pyrethroids							
Allethrin	25.60	123.0; 81.2	7	123; 79.1	22		
Bifenthrin	31.68	180.9; 166.1	12	180.9; 165.2	30		
Cyfluthrin	35.89	162.9; 91.1	15	162.9; 127.1	5		
Cypermethrin	36.64	180.9; 152.1	28	180.9; 127.1	30		
Deltamethrin	41.89	180.9; 152.2	25	253.0; 93.0	20		
Fenpropathrin	31.87	180.9; 152.1	27	181.1; 127.1	35		
Fluvalinate, tau	34.98	250; 200	40	250.0; 55.0	40		
lambda-Cyhalothrin	30.24	181.1; 152	25	197.0; 161.0	5	209.0; 111.0	15
Fenvalerate	39.24	166.9; 125.1	10	125.1; 89.1	25		
Permethrin	34.89	183.1; 168.1	15	183.1; 153.1	15	183; 115.2	25
Resmethrin	28.05	123.0; 81.4	10	123.0; 95.0	10		
Tetramethrin	31.67	164.0; 107.3	12	164; 135.1	10		
Carbamates							
Carbaryl	21.21	143.9; 115.1	28	143.9; 116.2	13		
Carbofuran	13.15	164.0; 103.0	25	164.0; 149.1	10		
Furathiocarb	32.49	163.0; 107.0	10	163; 77	30	164; 149.2	10
Pirimicarb	19.83	238.0; 166.2	7	166.2; 95.9	15		
Seven PCB congeners							
PCB 28	20.52	255.8, 186.1	28	257.8; 186	28		
PCB 52	22.48	291.9; 222.0	30	291.9; 220	30		
PCB 101	26.42	325.8; 256.1	39	325.8; 291	12		
PCB 118	28.67	325.7; 256.0	30	325.7; 254	27		
PCB 138	30.27	359.7; 289.9	30	359.7; 324.9	15		
PCB 153	29.40	359.7; 289.9	30	359.7; 287.9	30		
PCB 180	32.23	395.6; 325.9	30	395.6; 360.9	15		

* CE—collision energy.

**Table 2:** Validation parameters of the method.

Compound	LOQ ^a^ (μg/kg)	Recovery (%)	RSD ^b^ (%)	Linearity Range	r^2^
Organochlorine pesticides (OCPs)					
Aldrin	1	83.7	11.2	1–200	0.9967
Chlordane, cis-	1	94.3	13.5	1–200	0.9960
Chlordane, trans-	1	96.8	9.43	1–200	0.9983
Chlorobenzilate	1	85.4	7.19	1–200	0.9946
p,p′-DDD	1	104.2	17.4	1–200	0.9945
p,p′-DDE	1	107.3	11.1	1–200	0.9972
o,p′-DDT	1	104.7	16.5	1–200	0.9943
p,p′-DDT	1	101.3	8.41	1–200	0.9947
Dieldrin	1	92.2	16.2	1–200	0.9981
alpha-endosulfan	1	84.2	17.6	1–200	0.9954
beta-endosulfan	1	80.6	15.4	1–200	0.9975
endosulfansulfate	1	85.7	14.4	1–200	0.9969
Endrin	0.5	87.4	15.4	0.5–200	0.9948
alpha-HCH	0.5	99.3	11.6	0.5–200	0.9971
beta-HCH	0.5	98.6	9.77	0.5–200	0.9920
gamma-HCH/Lindan	0.5	97.4	10.4	0.5–200	0.9973
Heptachlor	1	94.6	15.1	1–200	0.9946
Heptachlorepoxid, egzo-	1	96.6	13.6	1–200	0.9947
Heptachlorepoxid, endo-	1	97.4	11.4	1–200	0.9978
Hexachlorobenzene (HCB)	0.5	91.3	16.7	0.5–200	0.9989
Methoxychlor	1	105.4	16.6	1–200	0.9930
Pentachloroaniline	1	106.9	14.5	1–200	0.9961
Quintozene	1	91.2	9.55	1–200	0.9926
Organophosphorus pesticides (OPPs)					
Azinphos-ethyl	2	87.3	11.2	2–200	0.9962
Bromophos-ethyl	1	101.4	18.3	1–200	0.9941
Carbophenothion	5	92.1	13.1	5–200	0.9929
Chlorfenvinphos	1	89.7	12.3	1–200	0.9983
Chlorpyrifos	1	87.9	11.6	1–200	0.9940
Chlorpyrifos-methyl	1	96.3	16.3	1–200	0.9950
Diazinon	1	94.7	15.6	1–200	0.9986
Dichlorvos	1	93.3	16.6	1–200	0.9975
Ethion	1	83.7	21.4	1–200	0.9905
Fenchlorphos	1	82.6	11.6	1–200	0.9943
Fenithrotion	1	88.0	8.87	1–200	0.9926
Fenthion	1	95.3	14.4	1–200	0.9940
Malaoxon	1	92.7	13.6	1–200	0.9945
Malathion	1	92.6	12.5	1–200	0.9930
Methidathion	1	96.9	15.4	1–200	0.9913
Mevinphos	1	107.7	14.7	1–200	0.9986
Paraoxon-metyl	5	118.4	13.3	5–200	0.9924
Parathion-ethyl	2	105.6	10.7	2–200	0.9908
Parathion-methyl	2	104.2	9.51	2–200	0.9981
Pirimiphos-methyl	1	86.4	13.9	1–200	0.9938
Profenofos	1	87.6	17.7	1–200	0.9947
Propetamphos	1	96.0	16.2	1–200	0.9951
Pyrazophos	1	105.7	12.2	1–200	0.9971
Tetrachlorvinphos	1	104.4	13.6	1–200	0.9920
Triazophos	1	88.7	15.3	1–200	0.9921
Pyrethroids					
Allethrin	2	98.6	7.51	2–200	0.9985
Bifenthrin	1	105.2	16.3	1–200	0.9963
Cyfluthrin	5	93.6	18.7	5–200	0.9918
Cypermethrin	10	105.7	11.6	10–200	0.9942
Deltamethrin	10	98.7	11.7	10–200	0.9954
Fenpropathrin	5	96.4	15.5	5–200	0.9926
Fenvalerate	5	96.4	12.4	5–200	0.9957
Fluvalinate, tau	5	97.2	9.22	5–200	0.9963
lambda-Cyhalothrin	5	90.7	15.4	5–200	0.9978
Permethrin	1	107.3	16.3	1–200	0.9974
Resmethrin	5	96.6	12.6	5–200	0.9906
Tetramethrin	1	97.9	16.2	1–200	0.9958
Carbamates					
Carbaryl	5	84.1	19.5	5–200	0.9911
Carbofuran	1	86.1	18.4	1–200	0.9923
Furathiocarb	1	98.6	8.55	1–200	0.9902
Pirimicarb	1	89.6	8.34	1–200	0.9952
Polychlorobiphenyls (Seven PCBs)					
PCB 28	0.5	102.4	8.43	0.5–200	0.9961
PCB 52	0.5	104.3	16.1	0.5–200	0.9983
PCB 101	0.5	94.6	10.4	0.5–200	0.9973
PCB 118	0.5	95.3	8.32	0.5–200	0.9965
PCB 138	1	97.8	10.6	1–200	0.9978
PCB 153	0.5	86.4	7.63	0.5–200	0.9964
PCB 180	1	84.3	10.3	1–200	0.9970

^a^ LOQ—limit of quantification; ^b^ RSD—precision in case of repeatability.

**Table 3:** Mean concentrations and minimum–maximum range (μg/kg, fresh weight) of pesticides and 7 PCB congeners in cow’s, goat and sheep’s milk and dairy products collected in Croatia within the period 2015–2022 (other pesticides not listed in the table were not quantified above the LOQ (<LOQ).

Compound	Mean ± SD (Number of Results above the LOQ) Minimum–Maximum			
	Cow’s Milk *n* = 337	Goat’s Milk *n* = 60	Sheep’s Milk *n* = 46	Dairy Products *n* = 91
Organochlorine pesticides (OCPs)				
Chlorobenzilate	1.30 ± 0.17 (4) 1.2–1.6	1.25 ± 0.05 (2) 1.2–1.3	<LOQ	<LOQ
Chlordane, trans-	1.2	<LOQ	<LOQ	<LOQ
∑DDT ^a^	1.70 ± 0.82 (21) 1.0–3.9	2.30 ± 0.40 (2) 1.9–2.7	1.3	3.17 ± 2.15 (3) 1.0–6.1
Dieldrin	1.1	<LOQ	<LOQ	<LOQ
∑endosulfan ^b^	1.68 ± 0.43 (4) 1.0–2.2	1.7	2.2	1.95 ± 0.05 (2) 1.9–2.0
Endrin	1.0	<LOQ	<LOQ	<LOQ
∑HCH ^c^	1.04 ± 0.34 (9) 0.7–1.5	1.07 ± 0.05 (3) 1.0–1.1	0.5	<LOQ
Heptachlor	<LOQ	<LOQ	<LOQ	1.1 ± 0.1 (2) 1.0–1.2
Hexachlorobenzene	0.92 ± 0.22 (23) 0.2–1.2	1.70 ± 0.6 (2) 1.1–2.3	1.03 ± 0.19 (4) 0.8–1.1	1.28 ± 0.28 (4) 0.8–1.5
Methoxychlor	1.78 ± 0.35 (6) 1.1–2.2	1.1	1.1	<LOQ
Pentachloroaniline	<LOQ	<LOQ	1.1	<LOQ
Quintozene	<LOQ	1.9	<LOQ	<LOQ
Organophosphorus pesticides (OPPs)				
Bromophos-ethyl	1.35 ± 0.05 (2) 1.3–1.4	1.25 ± 0.05 (2) 1.2–1.3	<LOQ	2.27 ± 0.05 (3) 2.2–2.3
Chlorfenvinphos	1.3 ± 0.3 (2) 1.0–1.6	<LOQ	<LOQ	<LOQ
Chlorpyrifos	1.49 ± 0.18 (9) 1.3–1.8	1.2	1.8	<LOQ
Chlorpyrifos-methyl	<LOQ	<LOQ	<LOQ	<LOQ
Diazinon	<LOQ	<LOQ	3.65 ± 1.95 (2) 1.7–5.6	<LOQ
Ethion	2.2 ± 0.3 (2) 1.9–2.5	<LOQ	<LOQ	<LOQ
Pirimiphos-methyl	1.4 ± 0.37 (3) 1.0–1.9	1.65 ± 0.35 (2) 1.3–2.0	<LOQ	<LOQ
Pyrazophos	1.0	<LOQ	<LOQ	<LOQ
Triazophos	<LOQ	1.6	<LOQ	<LOQ
Pyrethroids and carbamates (P&C)				
Allethrin	<LOQ	3.3	<LOQ	<LOQ
Bifenthrin	1.4 ± 0.2 (2) 1.2–1.6	<LOQ	<LOQ	<LOQ
Carbaryl	17.4	<LOQ	17.4 ± 7.40 (3) 12.0–27.9	<LOQ
Primicarb	1.5	<LOQ	<LOQ	<LOQ
Permethrin	1.4	<LOQ	<LOQ	<LOQ
Resmethrin	<LOQ	7.6	<LOQ	<LOQ
Tetramethrin	1.5	<LOQ	<LOQ	<LOQ
Polychlorobiphenyls (Seven PCBs)				
∑PCBs ^d^	2.74 ± 5.42 (19) 1.0–25.6	1.93 ± 0.18 (2) 1.75–2.1	1.38 ± 0.48 (4) 1.0–2.2	2.85 ± 0.61 (3) 2.4–3.9
Total no. of results above LOQ				
Total no. of OCP (%)	70 (20.8)	12 (20.0)	9 (19.7)	11 (12.1)
Total no. of OPP (%)	19 (5.64)	6 (10.0)	3 (6.52)	3 (3.30)
Total no. of P&C (%)	6 (1.78)	2 (3.33)	3 (6.52)	0
Total no. of PCBs (%)	19 (5.63)	2 (3.33)	4 (6.67)	3 (3.30)
Total	114 (33.8)	22 (36.7)	19 (41.3)	17 (18.7)

^a^ ∑DDT: sum of pp′-DDD, pp′-DDE, op′-DDT and pp′-DDT. ^b^ ∑endosulfan: alpha-endosulfan, beta-endosulfan and endosulfansulfate. ^c^ ∑HCH: sum of the alpha, beta and gamma isomers. ^d^ ∑PCBs: sum of PCB 28, PCB 52, PCB 101, PCB 118, PCB 138, PCB 153 and PCB 180.

## Data Availability

The original contributions presented in the study are included in the article, further inquiries can be directed to the corresponding author.
